# Is the neglect of exercise in anorexia nervosa research a case of “running out” of ideas or do we need to take a “LEAP” of faith into the future?

**DOI:** 10.1186/s40337-017-0157-z

**Published:** 2017-07-26

**Authors:** Stephen Touyz, Phillipa Hay, Melissa Noetel

**Affiliations:** 1 School of Psychology, The University of Sydney, Sydney, Australia; 2 Translational Health Research Institute (THRI), School of Medicine, Western Sydney University, Sydney, Australia

## Editorial

There is now a general consensus that Anorexia Nervosa (AN) is a serious mental disorder [1], with adolescent females being at the most risk for developing this debilitating illness [2]. Although unhealthy exercise was documented in the earliest descriptions of the disorder [3], it remains a significant barrier to recovery [4]. There has been a dearth of research as to what is best practice to address over-exercise in both the inpatient and outpatient treatment of adolescents with AN [4]. Despite calls to the broader research community for more action (e.g., the Meyer, Taranis and Touyz (2008) article entitled: “Excessive exercise in the eating disorders: A need for less activity from patients and more from researchers”) [5], such appeals appear to have been largely ignored. The most recently published clinical practice guidelines for the treatment of eating disorders [6] emphasized how important it was for clinicians to pay specific attention to over-exercise when assessing their adolescent patients presenting with AN. However, without scientific evidence and agreement as to what constitutes best practice, no specific recommendations were forthcoming to guide clinicians in addressing this not uncommon presenting problem.

One might well come to the conclusion that the challenge to address over-exercise particularly amongst adolescent patients with AN remains a bridge too far. In order to do so, there would need to be some common ground amongst the specialists in the field as to how over-exercise should be defined. Could such a consensus be reached or would such an attempt fail through the absence of a research base? On this front at least, there has been some progress. With an array of terminology (e.g., “excessive exercise, “compulsive exercise”, “obligatory exercise”, “driven exercise”) describing various aspects of physical activity in clinical and research settings, Noetel and colleagues [4] sought to canvass opinions from the leading clinicians/scientists in the field of adolescent eating disorders to settle on one term to describe this phenomenon. The authors undertook a Delphi study to explore whether consensus could be reached in the terminology used to describe unhealthy exercise in adolescents with AN [4]. A strong contender was “compulsive exercise” as it best encapsulated the rigidity and highly driven urge to exercise characteristic of AN, and is generally associated with the inability to cease over-exercising regardless of the risk of harmful consequences. Furthermore, “compulsive exercise” has generally been considered to be the most theoretically and clinically sound construct of exercise observed in eating disordered samples [7]. Although “compulsive exercise” was the preferred term when addressing unhealthy exercise behavior in adolescent patients with AN, the expert panel were unable to reach consensus. Could this considered group of experts then at least unite on the somewhat vexed issue as to what constitutes best practice in treating compulsive exercise in AN? Consensus was a bridge too far, however, the panel did agree upon some treatment approaches for adolescents with AN [4]. There was a universal agreement that family based treatment (FBT), which has been supported by established practice guidelines, should be the treatment of choice for this clinical population. However, there remains little guidance in this or other treatment manuals as to how over-exercise should be addressed. Furthermore, there is scant attention given as to how parents can be supported to reduce excessive exercise in their adolescent children or ensure that their caloric intake is sufficient to mitigate against energy expended. Although psycho-education for the adolescent and their primary support persons, emotion regulation, distress tolerance strategies and behavioral planning were clear front runners in general, the complexity of the behavior was not being addressed and consensus was lacking for the management of extreme levels of unhealthy exercise.

Whilst writing this editorial, it was not possible to escape the fact that Sydney was experiencing one of its hottest heatwaves ever recorded. The temperature was nudging 40 degrees C (104 degrees F). Fortunately most of the inpatients in Sydney with AN were in air-conditioned hospital wards where the ambient temperature would be approximately half of that despite the fact that “keeping patients warm may prove to be a beneficial treatment option” in reducing over-exercise [8]. Carrera and colleagues go on to say, “Consistent with recent research with an analogous animal model of the disorder, our findings suggest that ambient temperature is a critical factor contributing to the expression of excessive physical activity levels in AN” [8]. Why then are not all adolescent eating disorder units kept at a higher temperature to reduce physical activity? The answer to this question may be a little more complicated than meets the eye. The first rule of medicine is do no harm (*primum non nocere*) and there is evidence to suggest that increasing ambient temperature may in fact contribute towards decreased appetite [9]. The complexities surrounding this vexed issue abound.

There may however be some light at the end of the tunnel. Cognitive Behaviour Therapy (CBT) in its enhanced form, namely CBT-E, has shown promise in addressing unhealthy exercise in adults with AN. CBT-E incorporates strategies and techniques that focus not only on motivational strategies to promote change but on monitoring exercise behavior, encouraging healthy levels of exercise engagement and more effectively dealing with mood intolerance [10, 11]. More recently, the first targeted intervention to specifically address over-exercise in AN was developed by adopting a CBT framework. The compuLsive Exercise Activity theraPy (LEAP) is a semi-structured and problem-oriented intervention that has at its essence the targeting of factors that maintain compulsive exercise behaviours [12]. The results to date have been promising in adult patients with AN as those who have completed it have reported improvements in avoidance and rule driven exercise behavior, less use of exercise as a weight control strategy and a more flexible approach (less rigidity) towards exercise in general [13]. Since there is now emerging evidence that interventions that specifically target over-exercise in patients with AN can be clinically effective in reducing this worrisome behavior (which is often the last symptom to resolve), there is an urgent need for researchers in the field to address this, particularly within an adolescent population.

To assist in facilitating further research into compulsive exercise and its management, the *Journal of Eating Disorders* will devote a special issue to this issue in patients with AN edited by Professor Stephen Touyz (University of Sydney), Professor Phillipa Hay (Western Sydney University) and Professor Caroline Meyer (Warwick University).The proposed title of this special issue is “Raising the bar in the treatment of over-exercise in people with AN” with an anticipated publication date of November 2017. No longer should exercise be the “Cinderella” of AN phenomenology.
